# A Systematic Review of Psychometric Validation for Subjective Tinnitus Outcome Measures Assessing Acute Treatment Effects.

**DOI:** 10.1097/ONO.0000000000000067

**Published:** 2025-03-24

**Authors:** Julia Telischi, Jackson Rossborough, Brianna Kuzbyt, Suhrud M. Rajguru, Hillary A. Snapp, Tricia Scaglione

**Affiliations:** 1 Department of Otolaryngology, University of Miami Miller School of Medicine, Miami, FL; 2 Department of Biomedical Engineering, University of Miami College of Engineering, Miami, FL; 3 RestorEar Devices, LLC, Bozeman, MT; 4 Holistic Hearing & Wellness, LLC, Plantation, FL.

**Keywords:** Acute, minimum clinically important difference, Psychometrics, Questionnaires, Tinnitus

## Abstract

**Purpose:**

Tinnitus treatments are often scientifically evaluated using self-report questionnaires as primary outcome measures. However, guidelines for the appropriate application of these tools in research study designs are limited. This study aims to determine if any tinnitus outcome measure is validated for use in determining symptom change in response to treatments administered with hyperacute follow-up (less than 5 days).

**Databases Reviewed:**

PubMed, Embase, and Web of Science.

**Methods:**

A literature review was conducted of peer-reviewed articles on the psychometric properties of tinnitus outcome measures. A total of 594 articles were identified with 7 articles included for final review. Psychometric data, including the minimum clinically important difference and the time from intervention to outcome measurement (data collection interval), were extracted.

**Results:**

The final review included 5 studies on the Tinnitus Functional Index and 2 on the Tinnitus Handicap Inventory. The time intervals for intervention follow-up were defined as: 0–1 days = immediate, 2–5 days = hyperacute, 6–10 days = acute, 11–31 days = subacute, and >31 days = chronic. Two studies utilized chronic time intervals and 4 studies had follow-up in the subacute interval. The final study reported a wide follow-up range from hyperacute to subacute. No studies evaluated psychometrics with an immediate interval.

**Conclusion:**

There is no psychometrically validated tinnitus outcome measure for immediate treatment intervals and little evidence for hyperacute or acute intervals. Further research into the validity of tinnitus measurement tools in various time frames is required for the guidance of future study design.

Tinnitus is a perceptual auditory disturbance afflicting approximately 30 million people in the United States, or about 10% of the entire population (1). It has further been reported that 22.2% of people suffering from tinnitus experience significant distress described as disabling or nearly disabling to their quality of life (1). Tinnitus is generally defined as the presence of a ringing or buzzing noise in the ears that is typically heightened in quiet settings (2). The psychosocial impact of tinnitus on overall health is broad, including negatively affecting sleep and increasing stress and anxiety (3). Various treatment approaches have been explored to address tinnitus, including pharmacologics (4–6), sound therapies (7,8), cognitive-behavioral therapies (9,10), and electrical stimulation (11,12). Despite significant efforts and a large population of people affected by tinnitus, there are few treatment options available.

Part of the challenge in evaluating the efficacy of treatments for tinnitus is the current lack of objective measures (13). The primary method for determining the effectiveness of these treatments has largely relied on subjective measurement tools (ie, self-report questionnaires). This creates challenges in measuring clinically relevant changes with treatment. Subjective tools rely on patient perception, which can vary widely between individuals, while the multifactorial and subjective nature of patients’ experience with tinnitus can lead to inconsistent responses (14). Additionally, the subtle changes in a patient’s condition may not be perceived or reported accurately making it harder to detect small but clinically significant differences. Nonetheless, several subjective measurement tools have been developed to evaluate how those who suffer from tinnitus perceive their symptoms. Hall et al (15) identified in the literature 78 different self-report instruments used to assess tinnitus. While many of these scientific survey tools are validated for diagnosis and research, their validation processes have differed significantly and may not be applicable to every potential application for the instrument. Further, the wide variability in how these instruments are applied contributes to inconsistencies in study design across the field. This variability presents a critical challenge for any new research trial: determining which subjective measurement tool is best suited for the specific study design and objectives? Selecting the right survey is essential to ensure that the data collected are accurate and meaningful and that the results can be reliably interpreted within the context of one’s research objectives.

The most common subjective tools used for tinnitus research include questionnaires such as the Tinnitus Handicap Inventory (THI), Tinnitus Functional Index (TFI), Tinnitus Questionnaire, Tinnitus Handicap Questionnaire, Tinnitus Severity Index, and the Tinnitus Reaction Questionnaire (15). These questionnaires often categorize questions under subscales to capture multiple domains of the tinnitus experience, such as auditory perception, psychological/emotional effects, sleep, impact on lifestyle, and health effects (16). Not every domain in these questionnaires may be applicable to a treatment of interest. In such cases, simplified subjective tools, such as visual analog scales (VAS) or the Clinical Global Impression scale (CGIS), have been implemented (17). When using VAS and CGIS subjective tools, researchers design custom-made sets of questions unique for a particular research question (ie, “Please rate the total improvement of your tinnitus complaints compared to before the beginning of treatment.”) (18). Responses are typically designed as numeric-like scales with custom anchors (ie, always, sometimes, never). CGIS allows patients to provide an evaluation of their tinnitus as a whole rather than broken down into more specific domains as is done by the longer validated questionnaires. In VAS-based question sets, researchers often select a small number of tinnitus experience components that are of specific interest to their study design. Though these methods are frequently used (often as a secondary outcome measure in combination with a validated questionnaire) (15), there is little data on the validity of this approach and the significance and utility of any results for broader application of the treatment or therapy at hand. Hall et al (15) have thoroughly reported on the use of such subjective tools and the need for stronger guidelines on the study design of tinnitus research. However, there is still a lack of consensus on study design for tinnitus research, especially for acute treatments.

Most tinnitus research and treatments focus on extended intervention periods and/or long-term improvement over time (19). In accordance with this, questionnaires have historically been designed to survey improvement over weeks, months, or years. Such tools, however, may not be appropriate to accurately measure acute changes in tinnitus in response to treatment. Moreover, this can limit a researcher’s ability to define clinical significance when measuring change in response to treatment, influencing the analysis of outcomes as a whole. Neuromodulation and pharmacologic treatments aimed at immediate tinnitus relief are on the rise. As the field of tinnitus research expands to include new treatments, valid measures able to detect clinically meaningful change in more acute intervals will be increasingly important. The psychometric properties and validation of subjective tinnitus instruments over varying treatment intervals should be taken into account when considering their use in determining the effectiveness of treatment. In the present study, we aimed to identify the current subjective instruments used to measure change in tinnitus, evaluate the psychometric properties of these measures, and assess their ability to detect clinically significant changes over immediate to acute treatment intervals (1–10 days).

## METHODS

A systematic review was conducted to identify and analyze the psychometric properties of subjective tinnitus instruments used to determine changes in perception of tinnitus in response to treatment to assess their ability to detect clinically significant changes over immediate to acute treatment intervals (0–10 days). A literature search was conducted using PubMed, Web of Science, and Embase, using combinations of the key terms “Tinnitus,” “Surveys and Questionnaires,” “Patient Reported Outcome Measures,” “Psychometrics,” “Reproducibility of Results,” “Minimum Clinically Important Difference,” and “Validity” (Table 1). The database search was completed on May 18, 2022, and included all research articles published up to May 2022. The search was slightly modified to make use of database search features. The full search input for each database can be seen in Table 1. The terms “validity” and “minimum clinically important difference (MCID)” were determined by an initial review of articles evaluating the psychometric properties of tinnitus questionnaires to select studies that specifically reviewed the intrinsic ability of questionnaires to determine clinically significant change for research purposes. “Reproducibility of results” was also included as some articles may report MCID as a statistical result of a reproducibility study. The full text of the articles was accessed via each database’s own search engine.

**TABLE 1.** Search strategy and terms used

Database	Syntax	Result
PubMed	((“Tinnitus”[Mesh] OR Tinnitus) AND (“Surveys and Questionnaires”[Mesh] OR “Patient Reported Outcome Measures”[Mesh] OR Patient-Reported Outcome Measures OR Survey* OR Questionnaire* OR Screen* OR VAS OR “Visual Analog Scale”)) AND (“Psychometrics”[Mesh] OR “Reproducibility of Results”[Mesh] OR Psychometrics OR Reproducibility of Results OR Convergent Validity OR Minimum clinically important change OR MCID OR “validity” OR “validation”)	337
Web of Science	ALL = (Tinnitus AND (Patient-Reported Outcome Measures OR Survey* OR Questionnaire* OR Screen* OR VAS OR “Visual Analog Scale”) AND (Psychometrics OR Reproducibility of Results OR Convergent Validity OR Minimum clinically important change OR MCID OR “validity” OR “validation”))	308
Embase	(“tinnitus”/exp OR tinnitus) AND ((“patient”/exp OR patient) AND reported AND (“outcome”/exp OR outcome) AND measures OR survey* OR questionnaire* OR screen* OR vas OR “visual analog scale”/exp OR “visual analog scale”) AND ((((“psychometrics”/exp OR psychometrics OR “reproducibility”/exp OR reproducibility) AND of AND results OR convergent) AND (“validity”/exp OR validity) OR minimum) AND clinically AND important AND (“change”/exp OR change) OR mcid OR “validity”/exp OR “validity” OR “validation”/exp OR “validation”)	261

The selection of eligible publications was carried out according to the Preferred Reporting Items for Systematic Reviews and Meta-Analyses 2020 checklist (20).

An initial screening of the abstracts was performed to exclude any studies that were not peer-reviewed, studies in a non-English language, and studies using a non-English version of the questionnaires to reduce confounding bias.

**FIG. 1.**
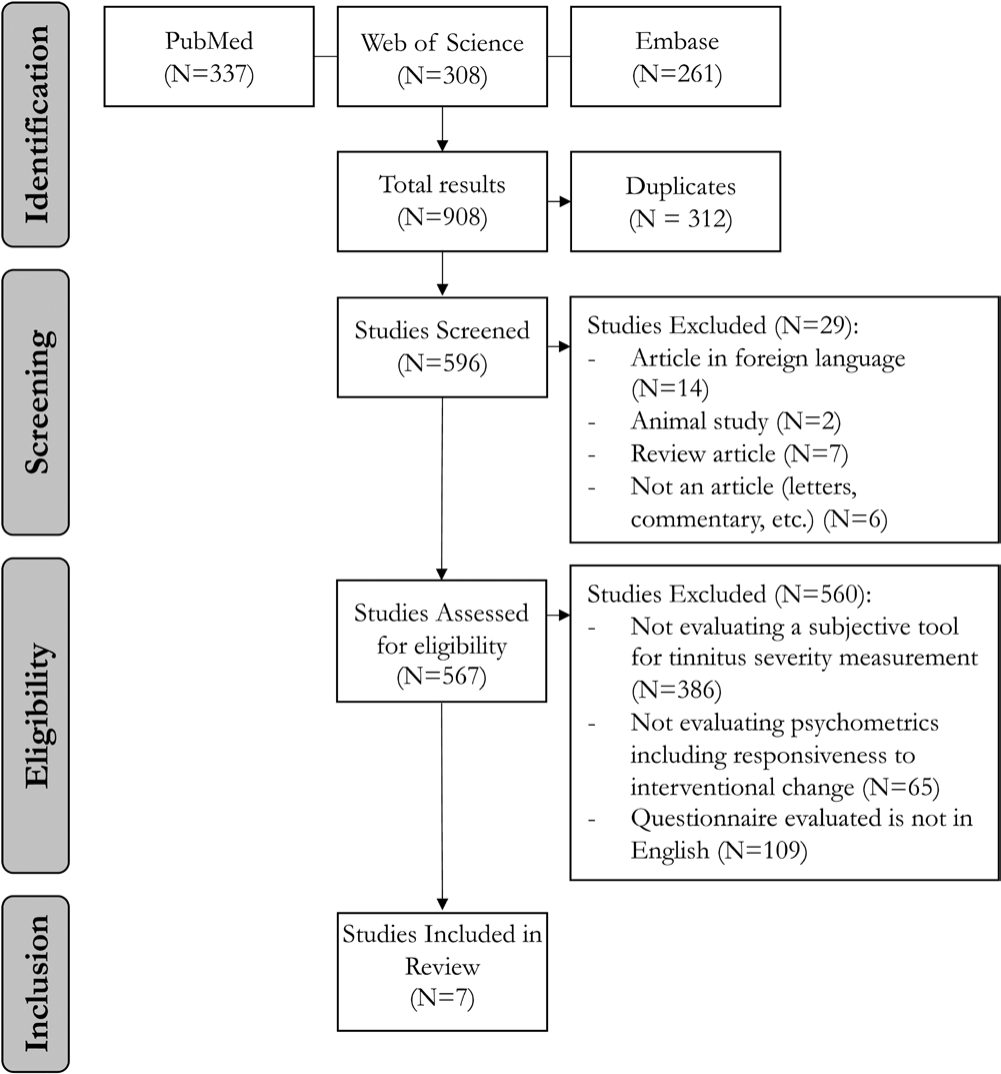
PRISMA flow diagram with inclusion and exclusion criteria of study selection. PRISMA, Preferred Reporting Items for Systematic Reviews and Meta-Analyses

Articles were then screened for relevance based on titles and abstracts by 2 independent reviewers and 1 senior scientist. Articles were excluded if they contained research where the keywords were not used according to the focus of our study, such as studies that did not evaluate a subjective tool to measure tinnitus severity or report on the psychometric properties of the tool (Fig. 1). Nonrandomized studies such as case reviews, letters, book chapters, and communications were also excluded. There were no restrictions placed on the time frame of this review. The eligibility criteria were then applied to the remaining abstracts. Any disagreements or questionable article inclusions were resolved by a third reviewer.

Following this process, the full text of the remaining articles was assessed for inclusion. Peer-reviewed randomized controlled trials assessing the psychometric properties of subjective tinnitus evaluation tools were considered for review. Only studies with tinnitus as the primary patient complaint were included. All types of tinnitus questionnaires were eligible for review, including those focusing on specific domains, such as quality of life, annoyance, or loudness.

In accordance with the study's aim to assess the ability of tinnitus questionnaires to detect interventional change, articles were further reviewed for inclusion based on which psychometric properties were analyzed. Only articles that reported a measurement of responsiveness to interventional change (most often represented as MCID/MCIDs/change) as a psychometric outcome were included. Studies using both distribution-based and anchoring-based methods of measuring psychometric responsiveness were acceptable.

Upon application of the eligibility criteria, the final studies were reviewed with manual data extraction for the following parameters: number of subjects, questionnaire used, intervention, MCID, MCID measurement method, and time of survey postintervention (data collection interval). Detecting changes in tinnitus over shorter periods of time was of particular interest for the purpose of this review. Therefore, data collection intervals were categorized as follows: 0–1 days = immediate, 2–5 days = hyperacute, 6–10 days = acute, 11–31 days = subacute, >31 days = chronic. Other psychometric properties including reliability and validity reported in the included studies were also recorded.

## RESULTS

### Study Selection

According to the search strategy described in Figure 1, 908 records were identified. After titles and abstracts were screened for eligibility, a total of 7 studies underwent full-text review. The majority of studies excluded (N = 368) did not evaluate the properties of subjective tinnitus measurement tools, followed by studies reporting on foreign language translations of the questionnaires (N = 109). After a thorough examination, 7 studies were included for review.

### Study Characteristics and Data

Summary of the studies included is reported in Table 2.

**TABLE 2.** Included study characteristics

Authors	Title	Journal	Publication Year	# Subjects	Questionnaire Studied Treatment Type^a^	Effect Size	MCID	MCID calculation method	Data collection interval category	Data collection interval
Hall et al (21).	How to choose between measures of tinnitus loudness for clinical research? A report on the reliability and validity of an investigator-administered test and a patient-reported measure using baseline data collected in a phase IIa drug trial	*Am J Audiol*	2017	91	Tinnitus Loudness Rating (Q2 of Tinnitus Functional Index [TFI])	n/a	3.5	Distribution	Mixed	3–19 days; 20–35 days
Fackrell et al (22).	Psychometric properties of the Tinnitus Functional Index (TFI): Assessment in a UK research volunteer population	*Hear Res*	2016	100	TFI	n/a	22.4	Distribution	Subacute	15 days
Chandra et al (23).	Psychometric validity, reliability, and responsiveness of the Tinnitus Functional Index	*Am Acad Audiol*	2018	40	TFI	n/a	4.38	Distribution	Subacute	14 days
Henry et. al (24).	Tinnitus Functional Index: development, validation, outcomes research, and clinical application	*Hear Res*	2016	167	TFI “Telephone tinnitus education”	1.23 (TFI) 1.04 (Tinnitus Handicap Inventory [THI])	n/a	Anchoring	Chronic	6 months
Meikle et. al (25).	The tinnitus functional index: development of a new clinical measure for chronic, intrusive tinnitus	*Ear Hear*	2012	128	TFI “Standard clinical treatment”	3 months: 0.83 (TFI) 0.56 (THI) 0.83 (VAS-severity) 6 months: 1.46 (TFI) 1.22 (THI) 0.80 (VAS-severity)	13	Anchoring	Chronic	3 and 6 months
Newman et al (26).	Development and psychometric adequacy of the screening version of the tinnitus handicap inventory	*Otol Neurotol*	2008	33	THI-S	n/a	10	Distribution	Subacute	16 days
Newman et al (27).	Psychometric adequacy of the Tinnitus Handicap Inventory (THI) for evaluating treatment outcome	*Am Acad Audiol*	1998	29	THI	n/a	20	Distribution	Subacute	20 days

a If applicable.

MCID, minimum clinically important difference; VAS, visual analog scale.

#### Tinnitus Questionnaires

All measures were self-report questionnaires. Only 2 tinnitus instruments were reported on in the included studies meeting inclusion criteria. Five studies reported on the psychometric properties of the TFI and two studies on the THI. The TFI consists of 25 questions covering the following domains: intrusiveness, sense of control, cognitive interference, sleep disturbance, auditory interference, relaxation, quality of life, and emotional effect. Questions are answered on a 10-point scale, and the patient is asked to evaluate how aspects of their tinnitus have affected them over the previous week. The THI also consists of 25 questions with answer choices of “yes,” “sometimes,” or “no.” The questionnaire covers 3 domains: functional, emotional, and catastrophic.

Hall et al (21) reported on only the second question of the TFI, “Over the past week, how strong or loud was your tinnitus?”. Unfortunately, they did not provide any evidence on psychometric properties for any other components of the instrument. Newman et al (26) evaluated the psychometric properties of a short version of the THI survey (THI-S), not the complete questionnaire. We could not identify any studies reporting on other English language tinnitus measurement tools.

#### Study Protocol Characteristics

##### Study Intervals

To measure the responsiveness of the questionnaire to changes in tinnitus, data had to have been collected from participants at more than one-time point. Of the 7 included studies, 6 collected data at 2 time points (1 baseline and 1 delayed measurement), and 1 collected data at 3 time points (1 baseline and 2 delayed measurements). A majority of studies (N = 4) utilized data collection intervals in the subacute range (11–31 days), with 2 studies reporting in the chronic (>31 days) range. Hall et al (21) evaluated a cohort of patients across a broad time range of 3–35 days covering the hyperacute (2–5 days), acute (6–10 days), and subacute (11–31 days) intervals (21). Patients in this study were tested at baseline and at 1 delayed time point, then retrospectively separated into 2 cohorts (3–19 days and 20–35 days) for analysis. While this could potentially include the hyperacute and acute phases, the study did not specifically report or analyze findings related to these distinct intervals. Therefore, no studies to date specifically address or provide targeted analysis on the immediate, hyperacute, and acute phases within this follow-up period.

##### Interventions

Two of the 7 studies used data collected from patients currently receiving intervention for their tinnitus. Meikle et al (25) treated the patients in their study with “standard clinical treatment”; there was not a singular specified treatment regimen as data were collected from patients at multiple sites, with each site reporting data on their institutional standard of care treatment(s). Treatments used in the study included hearing aids, sound therapy, medical therapy, psychological treatment, relaxation training, cognitive-behavioral training, and education. Henry et al (24) examined treatment with telephone-based tinnitus education. The remaining 5 studies were not interventional and instead relied on the distribution of patient scores over two time points to estimate the minimum change in score that could be statistically significant. There were no studies identified that reported on MCID in response to a controlled and specific treatment, therapeutic, or intervention.

#### Psychometric Properties

The full reporting of psychometric properties for the included studies are presented in Supplemental Appendix 1, http://links.lww.com/ONO/A34.

##### Responsiveness

The psychometric property of highest interest for the purpose of this review was the responsiveness of tinnitus to treatment. Outcome measures for responsiveness were most frequently represented as MCID (6/7 studies), although 2 studies also reported an effect size. Responsiveness can be determined using either the distribution technique with calculations based on statistical measurements such as standard deviation or the anchoring technique, where a comparison to an analogous measurement value (such as a lab value or patient-reported outcome) after intervention is used. The review revealed variance in methodology across the studies. In total, 5/7 of the included studies determined MCID using the distribution method, and 2/7 used anchoring.

##### Tinnitus Functional Index

Four of the 6 studies reported an MCID for the complete TFI questionnaire. A review of Table 2 highlighting included study characteristics, shows that the resulting MCID in total score for the TFI varied considerably, ranging anywhere from 4.8 to 22.4. Fackrell et al (22) used statistical analysis of the survey score distribution at 2-week data collection intervals (subacute range) to estimate that significant responsiveness to tinnitus symptom change would be a 22.4-point change in total TFI score, while Chandra et al (23) found an MCID of 4.38 with a similar method of statistical calculation and 2-week data collection interval (23). The large difference in the resulting MCID reported by these 2 studies highlights potential inconsistencies in using the distribution technique which may be related to differences in sample sizes, which were 100 and 40 for Fackrell *et al* (22) and Chandra *et al,* (23) respectively. Over longer data collection intervals of 3 months and 6 months (chronic range), Meikle et al (25) found MCID to be a 13-point change in total TFI score in response to “standard clinical therapy”. When looking at responsiveness for the tinnitus loudness rating question (Q2) of the TFI only, Hall et al (21) reported an MCID of 3.5. In their study, data collection intervals were pretreatment and not standardized, with MCID calculated from data collected at any timepoint within 3–35 days. Given this, their resulting MCID cannot be considered for acute responsiveness to treatment. Table 2 shows effect size calculations for responsiveness of the TFI with effect size increasing at later time points.

##### Tinnitus Handicap Inventory

MCID of the THI was calculated using the distribution method in 2/7 of the included studies. Newman et al (27) reported an MCID of 20 in a subacute data collection interval of 20 days. In a follow-up study, Newman et al (26) reported an MICD of 10 at a 16-day data collection interval for the abbreviated THI version, the THI-S. As with the TFI, Meikle et al (25) and Henry et al (24) reported increase in effect sizes for the THI at later time points (Table 2).

## DISCUSSION

Interventions for tinnitus have focused largely on the responsiveness of patients to chronic treatment. Objective tools and biomarkers to measure changes in response to new interventions are under study and include blood tests, electrophysiology, radiology, and/or balance tests. However, the challenges and heterogeneity of tinnitus presentation limit the validity and applicability of these methods for detecting meaningful changes for patients in acute or chronic periods. In literature and clinical studies, subjective tinnitus evaluation tools have been the primary focus for evaluating responsiveness to treatments over an acute time interval. As research on new treatment options for tinnitus expands, validated questionnaires for real-time responsiveness to each treatment will be increasingly important. The tools may need to be different for therapies aimed at immediate symptomatic relief versus those endeavoring to generate sustained or permanent changes in health status or symptoms. In this broad review, we did not identify a single appropriate subjective tool to measure acute responsiveness to treatment of tinnitus.

Our initial literature search identified 596 studies, demonstrating the broad use of tinnitus questionnaires in the literature. Only 7 (<2%) of those reported responsiveness, indicating a significant gap in the ability to measure and capture clinically relevant changes over various treatment intervals. Additionally, those included only covered 2 tinnitus evaluation tools, the THI and TFI, despite the availability of an array of evaluation tools, their wide use in clinics, and the even broader application of these tools across tinnitus research (28). Although both questionnaires were shown to be valid and reliable when they were created, this finding implies that they are now being widely applied to scenarios for which the appropriateness of their use is uncertain (25,29). The low number of studies meeting this review’s criteria can be attributed to lack of robust validation of the tools for more varied research protocol applications, or lack of focus on the ability to measure clinically significant change for patients. One possible explanation is that many of the tools were developed for clinical (evaluation) use so that providers could better understand individual patients’ baseline symptom levels and track changes in perception over time. However, these have subsequently been applied as a quantitative research tool. Nevertheless, this review provided key insights into how tinnitus instruments have been validated to evaluate treatment responsiveness or interventional change.

Owing to several factors, the responsiveness of the questionnaires was inconsistent between studies. Importantly, studies meeting inclusion criteria varied in how responsiveness was reported. The most frequently employed metric was the MCID, which indicates the score change necessary in a subjective tool to detect clinically noticeable changes for the patient. There are 2 commonly used methods for calculating MCID: distribution and anchoring (30). The anchoring method involves a comparison of questionnaire results to another value of interest, such as a lab value or a patient-reported outcome, while the distribution method uses statistical analysis (such as Standard Error of Measurement or Standard Deviation) of only the questionnaire results to determine an MCID (30).

Both methods of MCID calculation were used in the validation of TFI, but they did not result in a consistent MCID value across studies. The study that used the anchoring method found an MCID of 13 with treatment of “standard clinical intervention” (25). Of note, the study collected data at both 3 and 6 months and reported stability in the magnitude of treatment-related change at both time points (25). While both time points are considered here to be chronic treatment response times, this finding suggests that their reported MCID value may be relevant at shorter follow-up as well. The studies that used the distribution method resulted in MCID for TFI of 22.4, 10, and 4.8 with no specified interventions and a data collection timeline of 14–16 days or 3–35 days (a significantly shorter follow-up time than the studies using the anchoring method). The stark difference in MCID (22.4 vs 4.8 for (22) and (23) respectively) calculated at a similar time interval of 2 weeks is surprising. The major difference noted between these 2 studies is the number of subjects. Fackrell et al (22) evaluated 100 patients whereas Chandra et al (23) evaluated only 40 (22,23). This result shows that MCID may be affected by data collection interval, MCID calculation method, intervention type, or number of subjects in the study. The high variability in what change of TFI score constitutes a meaningful change for patients also may be due to the nature of the questionnaire itself. The TFI covers many domains and each patient experiences their tinnitus in an individual way. It is very likely that certain domains are more relevant for different patients and may not be broadly applicable to the population affected by tinnitus. Additionally, none of the included studies examined a specified, controlled treatment and how the application of this may affect MCID. Similarly, the reported MCID for the THI varied from 10 to 20 (Table 2). As such, a singular value for MCID in response to treatment cannot be defined for either the TFI or THI.

Lack of consistency across studies, in particular data collection intervals and intervention types, leads to conflicting outcomes on MCID. The data collection intervals used in each of the validation studies varied, although not as greatly as intervals in the literature (31–33). Generally, data in the validation studies was collected at approximately 2 weeks or at 3–6 months. No validation studies were found that utilized an immediate or hyperacute interval (ie, 0–5 days). However, treatments such as transcranial direct current stimulation or repetitive transcranial magnetic stimulation are commonly assessed acutely with intervals as short as less than 24 hours (31,33–37). Notably, the work by Hall et al (21) which evaluated only question 2 of the TFI (tinnitus loudness rating) included some patients in the hyperacute interval (2–5 days), suggesting that components of the THI may be useful for detecting changes in tinnitus in the hyperacute period. However, their analysis did not stratify patients across a wide follow-up interval (3–19 or 20–35 days) and did not describe the distribution of intervals in their patient cohorts, limiting the ability to draw definitive conclusions about responsiveness specifically within hyperacute intervals. Another method of examining responsiveness is to use effect size. Effect size is generally calculated by dividing the difference of 2 data set means by the standard deviation, so it does not take into account patient input on a change in their condition. It is therefore considered a less biased measurement than MCID (38). Henry et al (24) showed that the effect size captured by TFI was larger than the effect size of the THI for telephone tinnitus education intervention. The effect size of THI was also greater than that of THI at both 3 months and 6 months in the study by Meikle et al (25) after standard clinical treatment intervention. This suggests that the TFI is more sensitive to a change in tinnitus perception with treatment. Meikle et al (25) also showed that, unlike the MCID, there was an increase in effect size from the 3 to 6 month interval, which matches the expectation of greater change in tinnitus symptoms with a longer duration of treatment. However, its utility for measuring acute change remains unclear.

Although patients and providers may be interested in treatments that can offer immediate or short-term symptomatic relief, we find no evidence that any tinnitus instrument has been validated to assess treatment effects for immediate or hyperacute time periods. This review also finds that evidence is lacking to support how these tools are used over different time intervals. For example, tinnitus instruments are consistently used throughout the literature to measure changes in tinnitus over a broad range of time intervals, often for the purpose of measuring responsiveness to treatment. However, the assessment of psychometric properties of these instruments at differing time intervals has not been robustly studied. As an example, patient responsiveness to pharmacologic or neuromodulation treatments is expected to occur time-linked to the treatment or shortly thereafter administration of treatment. In such cases, differences would also be expected to dissipate over time as patients return to baseline status. Thus, recall or observation periods outside the treatment window would not be appropriate to detect changes. Clinicians and researchers should take methodological quality assessment into account when considering the ability of a given instrument to adequately measure MCID following treatment or over time. The evidence presented here supports the use of the TFI when measuring change over 2 weeks or 6 months or THI over approximately 1 month. However, care should be taken in considering the significance of changes seen according to the length of data collection intervals in each particular study. THI and TFI have been used to evaluate tinnitus response in as short as 5 days after treatment (33,35,39,40). There have been no studies examining the use of THI or TFI to evaluate acute response to treatment, and thus neither of these commonly used tools can be considered a valid measure of immediate posttreatment change in tinnitus.

The finding that TFI has not been validated for immediate or hyperacute treatment response intervals makes sense considering the content of the instrument. Questions are all specifically phrased so that the patient considers how various aspects of their tinnitus have affected them in the previous week. For example, question 2 of the tool states “Over the past week, how strong or loud was your tinnitus?” This innately precludes the tool from distinguishing rapid changes in patient condition. However, several of the domains assessed in the questionnaire, such as intrusiveness, cognitive interference, auditory interference, and emotional effect, could be relevant in a short time frame if the questions are rephrased. The THI, on the other hand, does not contain such direct verbiage for the duration of symptoms, although some questions imply consideration of symptoms over longer time periods. For example, question 1 states “Because of your tinnitus, is it difficult for you to concentrate?” which may be interpreted as a level of difficulty concentrating at a given moment. However, other questions such as “Do you complain a great deal about your tinnitus?” or “Does your tinnitus make it difficult for you to enjoy life?” require the patient to reflect on longer periods of time.

Most commonly, research protocols measuring acute changes in tinnitus in response to treatment use a combination of the questionnaires validated for a longer time frame and individually made VAS or CGIS questions (31,33–37). One version of a questionnaire using VAS in German has been validated on an acute timescale (17), but no similar studies were found for VAS questionnaires in English. Scale-based question styles such as VAS, numerical rating scale, or verbal rating scale have been studied more extensively in several other fields, such as clinical research in chronic pain (41). VAS has been shown in endometriosis pain to correlate most closely with CGIS and to be the most precise when compared to other question types (42). However, in other subsets of chronic pain, such as low back pain, no difference was found between the question styles (43). VAS is advantageous because it allows patients to select along a continuous gradient, and so they can give more variable responses compared to questions with discrete answer choices (such as a Likert scale) (44). However, disadvantages include patients sometimes encountering difficulty choosing the most representative location along the line, especially if they are not being asked to compare their current status to that at another timepoint (44,45). Across the literature of pain research, the VAS question in use is fairly standard, involving a 100 mm line with the qualifiers “no pain” and “worst pain” at each end (41). In other words, the same or similar question is applied for different diseases or sources of pain. This is in contrast to tinnitus research, for which different questions or qualifiers are used within 1 condition to determine slightly different outcomes (loudness vs annoyance vs disturbance, for example), leading to variability in question design across different studies. This review has found that despite their common use in quantifying tinnitus response, these question designs are not standardized and have not been adequately validated for application in tinnitus research.

The gap in validation of tinnitus questionnaires assessing MCID over immediate to acute time periods (0–10 days) creates a challenge in protocol development when researchers are evaluating new treatments. This has led to a diversity of measurement tools used and lack of consensus in testing methodology. Without consistent measurements, comparing the results of different studies becomes difficult, which ultimately may hinder the progression and cohesion of tinnitus treatment research.

## LIMITATIONS

There are several limitations of this review that must be acknowledged. First, only articles with questionnaires administered in English were included. Several relevant studies were not considered due to language restrictions. Search terms were selected with the goal of capturing all relevant papers. While not likely, studies may have been unintentionally missed.

## CONCLUSION

Collectively, the limited studies reporting on responsiveness do not provide full clarity or guidance for clinicians or researchers on how to reliably determine the effectiveness of treatment or how that might change acutely versus over time or sustained treatment. Investigators should carefully consider which assessment tools are appropriate for a specific research question or intervention. The array of available tools, their validity for capturing desired symptoms and outcome metrics, limitations with respect to subject size, and time points must be considered before initiating a study.

## ACKNOWLEDGMENTS

None declared.

## FUNDING SOURCES

This study was supported by U-LINK 21-1728 and the National Center For Advancing Translational Sciences of the National Institutes of Health UL1TR002736 to Miami CTSI (Snapp, HA), NIH 5R01DC019158 and VA 1I01RX003532 (Rajguru, SM).

## CONFLICT OF INTEREST

S.M.R. is the founder and Chief Scientific Officer of RestorEar Devices LLC. The company did not financially support the study. The other authors disclose no conflicts of interest.

## DATA AVAILABILITY STATEMENT

The data presented in this study are available upon request from the corresponding author.

## Supplementary Material


