# Supplementary material for: A Systematic Review of Psychometric Validation for Subjective Tinnitus Outcome Measures Assessing Acute Treatment Effects
Source: Otol Neurotol Open. 2025 Mar 24;5(1):e067. doi: 10.1097/ONO.0000000000000067 (PMC11949293; doi:10.1097/ONO.0000000000000067)
Supplement: Supplementary file 1 [file ono-5-e067-s001.pdf]

Author /year	DOI	Validity	Reliability	Reproducibility
Hall et all; 2017	10.1044/2017_AJA-16-0129	Convergent validity:<br>TLR to dB Hearing level loudness matching r = 0.35<br>TLR to dB sensation level loudness matching r = 0.29	Test-retest reliability (ICC) r = 0.66<br>SEM = 1.25<br>Limits of agreement = 2.61 (-2.54 - 2.78); 94%	n/a
Fackrell et al; 2016	10.1016/j.heares.2015.09.009	Construct validity<br>TFI and THI r = 0.82<br>TFI and THQ r = 0.82<br>TFI and VAS-L r = 0.46<br>TFI and PR-A r = 0.58<br>TFI and BDI r = 0.57<br>TFI and BAI r = 0.39<br>TFI and WHOQOL r = -0.48	Test-retest reliability (ICC) r = 0.91 (0.84–0.95)<br>Internal consistency α = 0.80	Internal consistency (α = 0.80);<br>Agreement 93%;
Chandra et al; 2018	10.3766/jaaa.16171	Convergent validity:<br>TFI to THQ r = 0.717<br>Divergent validity:<br>TFI to HHI r = 0.294	Test-retest reliability (ICC) r = 0.91<br>Internal consistency α = 0.97<br>SEM = 1.58	Internal consistency reliability from separate subject; cohort<br>Cronbach's alpha = 0.97
Henry et al; 2016	10.1016/j.heares.2015.06.004	n/a	n/a	n/a
Meikle et al; 2012	10.1097/AUD.0b013e31822f67c0	Convergent validity<br>TFI with THI r = 0.87<br>TFI with VAS r = 0.75<br>Discriminant validity<br>TFI with BDI-Primary Care r = 0.57	Test-retest reliability r = 0.78<br>Internal consistency α = 0.98	Cronbach's alpha = 0.97
Newman et al; 2008	10.1097/MAO.0b013e31816569c4	Correlation<br>THI to THI-S r = 0.90	Test-retest reliability r = 0.81<br>SEM = 4.1	n/a
Newman et al; 1998	No DOI available<br>Accession number: 9564679	n/a	Test-retest reliability = 0.92<br>SEM = 1.8-7.0	n/a

*THI = Tinnitus Handicap Inventory; TFI = Tinnitus Functional Index; HHI = Hearing Handicap Inventory; SEM = standard error of measurement; TLR = tinnitus likeness rating; ICC = intraclass correlation coefficient; THQ = Tinnitus Handicap Questionnaire; BDI = Beck's Depression Inventory; BAI = Beck's Anxiety Inventory; WHOQOL = World Health Organization Quality of Life; MCID = Minimum Clinically Important Difference; VAS = Visual Analogue Scale
